# Supplementary material for: High-resolution tracking of hyrax social interactions highlights nighttime drivers of animal sociality
Source: Commun Biol. 2022 Dec 15;5:1378. doi: 10.1038/s42003-022-04317-5 (PMC9755157; doi:10.1038/s42003-022-04317-5)
Supplement: Supplementary file 2 — Supplementary Information [file 42003_2022_4317_MOESM2_ESM.pdf]

Supplementary Information - High-resolution tracking of hyrax social interactions highlights nighttime drivers of animal sociality

Camille N. M. Bordes[1], Rosanne Beukeboom[1], Yael Goll[2], Lee Koren[1], Amiyaal Ilany[1*],

[1] Faculty of Life Sciences, Bar-Ilan University, Ramat Gan (Israel)
[2] School of Zoology, Tel Aviv University, Tel Aviv (Israel)
[*] corresponding author: amiyaal@gmail.com

Supplementary Methods

Data collection and sampling

This study is part of a long-term project initiated in 1999 at the Ein Gedi Nature Reserve in Israel (31° 28′ N, 35° 24′ E). The study area counts two distinct study sites – 35,437 and 10,286 square meters, respectively – within two neighbouring canyons. The two sites are located approximately 2.5 km apart. Between March and August 2017, the population of rock hyraxes in the study area counted 83 individuals (Supplementary Table 1), 41 of which were eligible for receiving proximity loggers.

Supplementary Table 1: Hyrax population in Ein Gedi (March-September 2017)

	Site 1	Site 2	Total
Pups [a]	14	9	23
Juveniles [b]	9	10	19
Adults [c]	16	13	29
Bachelors [d]	8	4	12

Total	47	36	83

[a] <1 year old, [b] <= 2 years old, [c] >2 year old resident male or female, permanently living in a group, [d] >2 year old males, living outside social groups.

By the beginning of June 2017, the start of the data collection period, several adult females had left the study sites, resulting in 37 adult hyraxes being eligible for proximity loggers. Out of the eligible individuals, 81% were fitted with a collar for 2 months between June and August 2017 (Supplementary Table 2). During this period, 2 collared hyraxes disappeared a few days only after receiving their collars and were then excluded from the study. After that, the composition of the hyrax population fitted with proximity loggers remained stable as no other individual neither disappeared nor lost its logger.

Supplementary Table 2: Aggregated counts of adult hyraxes eligible to receive a proximity logger (number of deployed loggers) (June-August 2017).

	Site 1	Site 2	Total
Females	9 (9)	10 (6)	19 (15)
Resident males	4 (4)	2 (1)	6 (5)
Bachelor males	8 (7)	4 (3)	12 (10)
Total	21 (20)	16 (10)	37 (30)

We filtered out data points that were irrelevant to our study goal. These included times when only some of the hyraxes were fitted with proximity loggers. Hence, we included in our analysis only proximity interactions occurring between July 14th and August 10th, 2017, resulting in a study period of 27 consecutive days.

Our goal was to study the structural differences between daytime and nighttime social networks. To do so, we defined which social interactions belong to daytime and which to

nighttime. We labeled as “daytime interactions” any interaction occurring between sunrise and sunset, in contrast to “nighttime contacts”, which we defined as interactions occurring when the sun was at least 12 degrees below the horizon (i.e., from nautical dusk until nautical dawn). Note that we intentionally discarded interactions timestamped between nautical dawn and sunrise on the one hand and sunset and nautical dusk on the other hand. Hyraxes display a transition in their social behavior during these two periods, when they bask in groups around their dens, either in anticipation of nightfall or prior to foraging in the morning. This behaviour makes them unlikely to interact with other individuals but their sleeping group and strongly biases their interaction pattern in favour of affiliates sharing their den. As a result, these raw proximity contacts could not be attributed to either daytime or nighttime datasets and were removed from our analysis.

Constructing proximity-based networks

Before analyzing time-aggregated social networks, we must pre-process the raw data returned by the collars. There are multiple ways to build a social network based on proximity loggers’ data. For instance, one can count the number of contacts between two individuals or compute the total amount of time they spent together over a study period. However, Watson-Haigh *et al.* (2012) reported important intra and inter-logger variability in device performance, with some pairs consistently failing to detect each other (hereafter ‘poor reciprocal agreement’), and a tendency of collars to record long interactions as a series of shorter encounters rather than a single contact (hereafter ‘record multiplicity’). Both biases combined make raw numbers of encounters and contact durations unreliable to estimate edge weight in proximity-based animal social networks. Instead, proximity data require data correction before performing social network analysis. As loggers’ performance in the laboratory can predict loggers’ performance on-field [2], we performed a series of tests under laboratory conditions to assess their quality before deployment.

In theory, proximity loggers record social encounters symmetrically and should display a perfect ‘reciprocal agreement’ (i.e., the percentage of overlap between reciprocal records of the two loggers in a dyad [2]). However, proximity loggers vary in their performance due to electronic noise and variation in individual position in space [3]. This bias was found likely to generate error in the structure of the related proximity network [2]. Therefore, our first concern was assessing the ‘reciprocal agreement’ or ‘reciprocity index’ between every possible pair of loggers to avoid deploying incompatible collars together. Loggers were left interacting by pairs at 30 cm, at the height of 20 cm, under controlled conditions in the laboratory for 30 minutes. The order of pairs being tested was randomized (forbidding the same collar to be tested twice in a row), and all pairs were tested under the same conditions. Each pair of collars was then characterized with a ‘reciprocity index’, which we used to build a network of collars reciprocity. Pairs of loggers that consistently performed better during the pre-tests were clustered together in the field using a community detection algorithm, whereas failing dyads were deployed in different study sites to minimize their chances of encounter.

We then moved to the ‘record multiplicity’ bias. The challenge of this correction is that we need to identify which records are multiple detections of the same social encounter (and must be merged). For a given collar, it is possible to determine the ‘least common interval’ of time separating two consecutive encounters with the same individual [4]. Proximity contacts that are separated by a period shorter than the ‘least common interval’ are then likely to be multiple records of the same social event [4]. In rock hyraxes, our field-based data showed that an interval of 240 seconds efficiently discriminates between distinct proximity contacts recorded by a device. For each logger, we merged multiple records of the same encounter based on this threshold.

We then removed short contacts of less than 10 seconds [3]. These contacts can have two origins: 1) when two animals stand at the edge of their loggers’ detection range, their collars

tend to record one or a series of isolated 1-second contacts [5]; 2) short records of less than 10 seconds, which can be either due to agonistic interactions (e.g., fights/threats) or unintended proximity (e.g., two animals temporally passing by each other because of local obstacles). Whether these short contacts are the result of one or the other, they are unlikely to represent positive interactions, whereas longer records represent active affiliation or spatial tolerance between individuals.

At this stage, we measured the level of reciprocity in the collar's patterns of detection (x = 54.42 ± 30.49%). For example, sometimes collar "A" reported an interaction with collar "B", while "B" did not record an interaction with "A". This difference between loggers may be attributed to relative positions of the hyraxes, small differences in the loggers' internal clocks (i.e., internal offsets), or residual differences in the transmitter and the receiver performance [2]. As loggers' reciprocity is an important component of the reliability of the structure of proximity-based social networks [2], we reduced the resolution to 5-minute intervals. Lowering the time-resolution should logically increase loggers' reciprocal agreement, thus making edge weight more reliable when building the social network. To do so, we divided our study period into intervals of five minutes, for which each dyad received a value of either 0 (no interaction during the interval) or 1 (the dyad did interact for at least 10 seconds during the interval). This procedure increased the average dyadic 'reciprocity index' to 69.16 ±25.84%.

Notably, 7 proximity loggers were either never retrieved or permanently damaged, resulting in the loss of the data they recorded. To correct for this problem, we removed duplicate proximity contacts from dyads where both collars were retrieved by randomly excluding the records from one of the loggers[6]. We repeated this random exclusion for each period of aggregation independently so that not always the same logger is removed from the dataset every day. As a post hoc verification, we also repeated our data analysis multiple times to ensure that our results were qualitatively robust to the subset of loggers retained by this random selection.

Proximity events were aggregated in various manners in this study to highlight variations in hyrax social structure over time. For each period of aggregation, we built weighted social networks using the simple ratio index[7] in the '*asnipe*' R package[8].

Discriminating between 'passive' and 'active' sociality at night

Sleep is characterized by a set of physiological states associated with lower levels of awareness [9], which affects individuals' likelihood to initiate interactions or end existing ones. When two individuals are awake and are in a long interaction, they may break the ongoing encounter at any moment. However, once animals are asleep, the contact lasts as long as both individuals remain unconscious. Consequently, social encounters recorded when two individuals are asleep are not the result of a repeated and active choice to remain near each other. Rather, they are the result of a social behaviour expressed while awake and being carried out after losing consciousness. Due to their length, 'passive' contacts have a strong impact on the social structure of an aggregated network which can cloud the 'active' sociality expressed in-between sleeping bouts. Since nighttime social structure is predominantly sleep-related in rock hyraxes, comparing social behaviours between daytime and nighttime requires ignoring sleeping associations. The proximity loggers we used do not collect physiological data [1], hence they cannot discriminate between behaviours. Yet, sleeping and resting present similarly low levels of behavioural activity [9], resulting in similarly long proximity contacts. Beyond a certain threshold of interaction length, it is thus reasonable to assume that two individuals are no longer actively maintaining their proximity but are rather engaged in either a resting or a sleeping bout. To determine which encounter length can discriminate between 'passive' and 'active' proximity contacts, we first aggregated all nocturnal social contacts into a nighttime network. We then filtered out interactions shorter than 5 minutes and built a social network based on the remaining interactions. We compared both networks using the cosine similarity index in the '*lsa*' R package [10]. We then increased the filtering criteria by steps of 5 minutes and built a series of

incrementally filtered networks until no interactions were left to filter out. We calculated the cosine similarity indexes between all possible pairs of filtered networks. In addition, we monitored the average individual strength centrality across subsets. If a threshold exists in interaction length after which hyraxes express 'passive' social encounters, we should observe a sudden drop in average strength centrality and cosine similarity indexes.

Neighbors' stability

At the individual level, we measured the proportion of social partners with which a hyrax keeps interacting between two consecutive periods and used it as a proxy for the stability of an individual immediate social environment. It is mathematically defined as the Jaccard index between its first-degree neighbors (i.e., any node linked by a direct tie independently of the tie weight) on the time-adjacent intervals P_1 and P_2. For an individual i interacting with its first-degree neighbours $N_1 = \{a, c, d, f\}$ over an interval P_1 (i.e., daytime on date n), and with its first-degree neighbours $N_2 = \{a, b, d, f, g\}$ over the interval P_2 (i.e., the following night):

$$J_i(N_1, N_2) = \frac{|N_1 \cap N_2|}{|N_1 \cup N_2|} = \frac{3}{6}$$

If V is the total number of individuals in the study population and n is the total number of time-intervals in our study period, for all individuals $i \in \{1, \dots, V\}$ and for all time-intervals $P_j \in \{P_1, \dots, P_{n-1}\}$, we measured the indexes $\{J_i(N_j, N_{j+1})\}$. We then assessed their individual significance using a permutation test for paired samples and combined all p-values into a global p-value using the competitive test implemented in the '*CombinePValue*' R package [11].

Group-level standard deviation in individual network traits

We calculated the standard deviation in individual strength centrality, degree centrality and eigenvector centrality at the group-level for every day and every night of the study period. We then compared them to scores calculated on datasets permuted 1,000 times according to the permutation procedure described in the main text (see *Permutation tests*). We detected a

Supplementary Results

Discriminating between ‘passive’ and ‘active’ sociality at night

When filtering out nocturnal social encounters by length, all networks predicted each other well (mean $r^2 \pm$ sd = 0.90 ±0.10). Yet, cosine similarity indexes clustered into two subgroups, separated by a threshold value of 25 minutes, and the average strength centrality decreased from 0.43 (±0.33) to 0.35 (±0.37) when removing interactions shorter than 25 minutes (Wilcoxon rank sum test: W=12,916, p=0.0005) (Supplementary Figure 1). This result shows that interactions shorter than 25 minutes and interactions longer than 25 minutes represent two different social structures. Similar results were observed for daytime contacts. Hereafter, we divide encounters into ‘passive’ (>25 minutes) and ‘active’ (<=25 minutes). Among proximity contacts recorded at night, about 44.6% were labelled as ‘passive’ vs. 20.1% during daytime. ‘Passive’ social encounters accounted for 30.2% of daytime and nighttime hyrax sociality combined.

We do not exclude that some ‘passive’ associations remain in the ‘active’ subset, or that long ‘active’ encounters were filtered out with the ‘passive’ subset. However, our results indicate that the proportion of residual ‘passive’ associations in our ‘active’ networks is too small to cloud the social structure arising from ‘active’ social behaviours, suggesting that 25 minutes is an adequate threshold to discriminate between these two types of interactions.

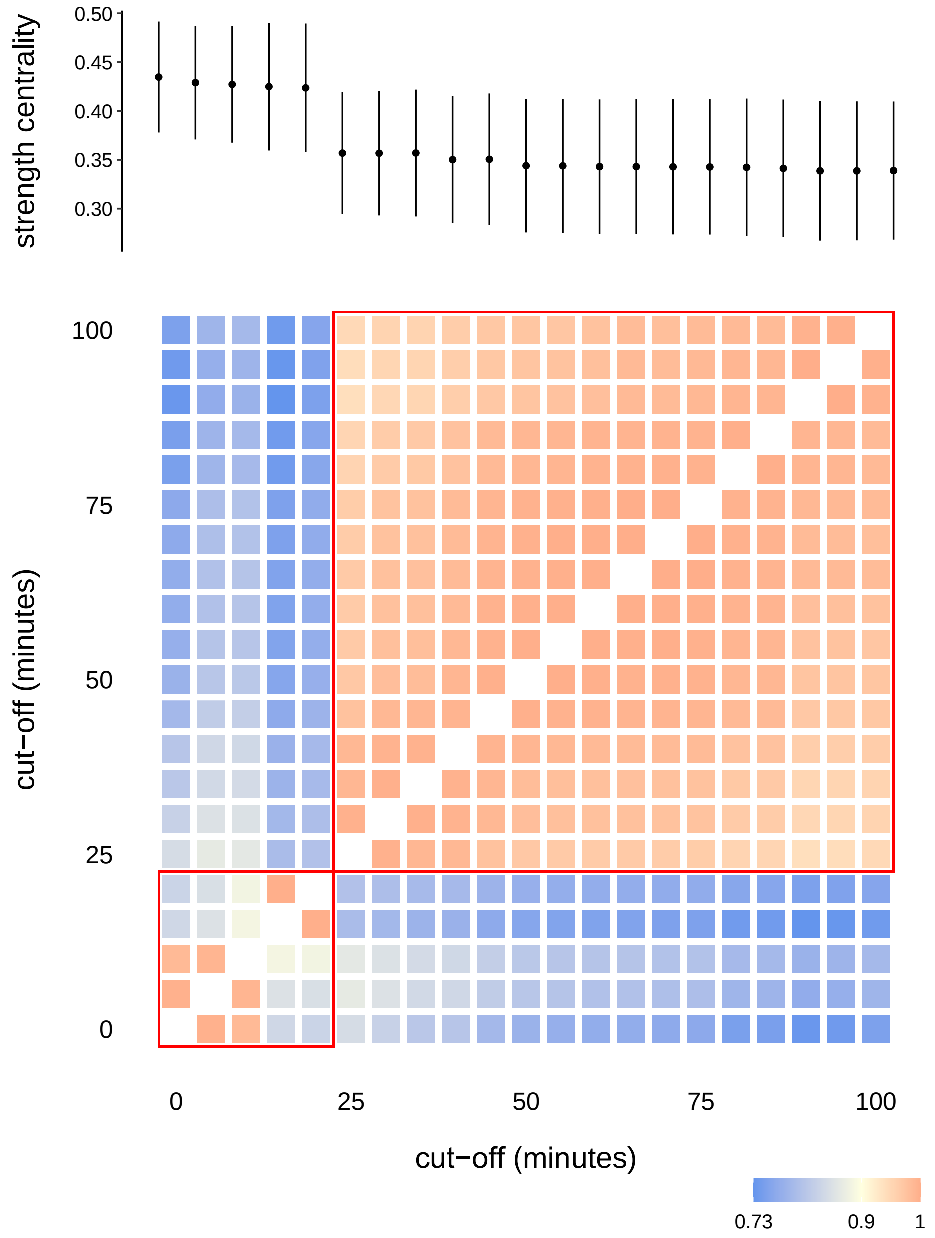

Supplementary Figure 1: Average strength centrality (top) and pairwise cosine similarity indexes (bottom) between time-aggregated networks based on interactions filtered by length (i.e., longer than the cut-off).

Neighbors' stability

As we expect hyraxes to spend most of their day with their social groups, we also expect individual immediate social environments to be stable (i.e., direct neighbors barely change over time), and the level of stability should be predicted under our null hypothesis (i.e., the permutation test should be non-significant). Jaccard indexes were globally higher than predicted under our null hypothesis (p<0.001), suggesting that most hyraxes maintain the identity of their direct neighbors more stable than expected by chance, and this pattern was repeated throughout the whole study period (Supplementary Figure 2).

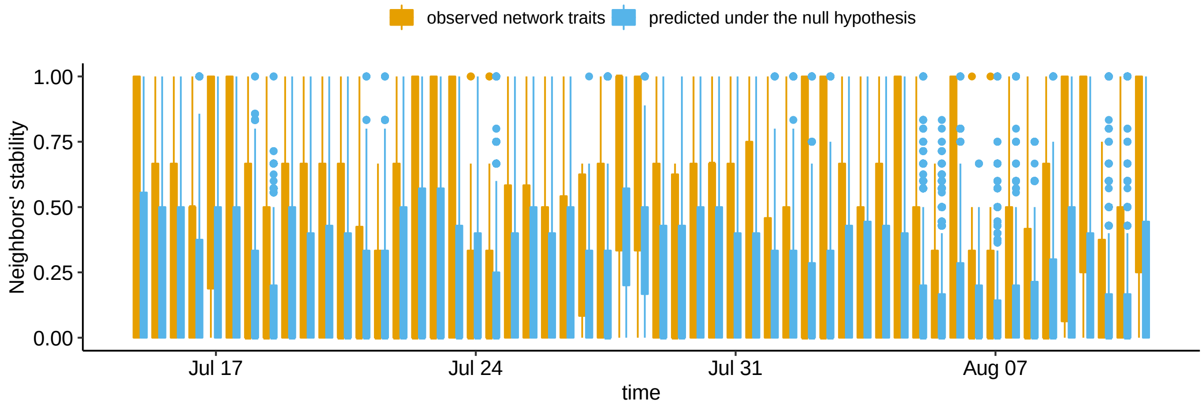

Supplementary Figure 2: Daily distribution of individual Jaccard indexes. Orange: Observed distribution of Jaccard indexes. Blue: Distribution that would be expected under the null hypothesis generated by the data-stream permutations.

Group-level standard deviation in individual network traits

We detected a significantly lower standard deviation in strength centrality and eigenvector centrality at night compared to daytime (p<0.001, Supplementary Figure 3), but not in degree centrality (p = 1, Supplementary Figure 3).

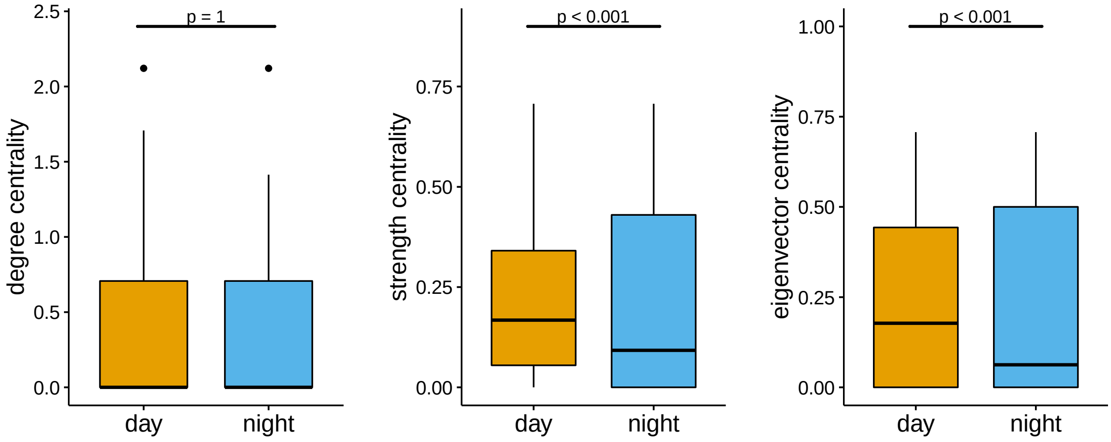

Supplementary Figure 3: Standard deviation in individual degree centrality (left), strength centrality (center), and eigenvector centrality (right) at night (blue boxplot) compared to daytime (orange boxplot).

Supplementary Discussion

When we divided the proximity contacts into 54 distinct time periods representing the days and the nights of the study period (27 days), we aggregated data over short periods thus producing relatively sparse social networks. This sparsity is partly due to structural zeros between individuals from different study sites. These zeros artificially increase the raw cosine similarity indexes reported in Figure 5 of the main text and requires that observed cosine indexes are interpreted in the light of the average level of correlations in the matrices of correlation. A second cause to network sparsity is the rarity of social interactions when networks are built over too short periods. Our study is likely to be affected as some nighttime networks count as little as about 60 proximity events between 28 individuals. When testing hypotheses on sparse networks, even small differences in animal social behaviours are likely to yield significant results in a permutation-based analysis. Here, we designed our permutations so that individual gregariousness is conserved over each period, meaning that asocial individuals maintain their non-sociality across permutations and that structural zeros are constant in all permutation-based comparisons. Paired with an FDR correction on permutation-

based p-values, this design reduces the risk of false positive in permutation-based comparisons in sparse animal networks. Unfortunately, it also leads to a substantial loss of statistical power when the permutations are only allowed within a very small subset of individuals.

In the results summarized in Figure 5 of the main text, we notice that weighted networks show higher correlation coefficients towards the end of the study period (Supplementary Figure 4) relatively to the rest of the matrix. This pattern is confirmed in the p-value matrix where the density of significantly high p-values increases towards the end of the study period. This result implies that networks became more similar to each other over the last few days of our study period. This pattern is however not repeated in the binary networks, suggesting that the difference in sociality occurs in edge weights more than edge position in the network. This change in weighted network structure could be the sign of the end of the mating season, when group members stabilize their association patterns after a period of increased sociality and between-group interactions.

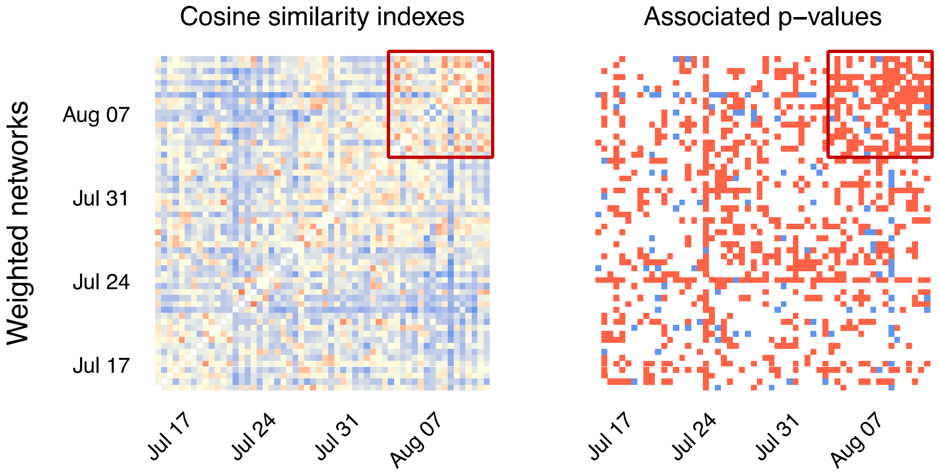

Supplementary Figure 4: Pairwise cosine similarity indexes between all possible pairs of daily weighted networks (left) and associated permutation-based (right). A red square is drawn on the region of the matrices where the raw cosine similarity indexes are higher than in the rest of

the matrix and the region of increased density of significant p-values showing that networks are more correlated than expected by chance.

Additionally, one day stands-out in the binary networks analysis which we identified this period as the night of the 7th of August 2017. It is characterized by relatively lower cosine similarity indexes with all the other days of the study period, a pattern again confirmed by the p-value matrix (Supplementary Figure 5). On that day, the network did not lose in density nor in transitivity, and the number of interactions and the placement of existing edges was not drastically different from other days (Supplementary Figure 6). However, we noticed that two social groups usually connecting between 4 and 6 individuals simultaneously disconnected into pairs for the night. Permutations predict these groups will interact every day, hence the structure of the observed network differed more than usual from the structure of the random network for one night, leading to very low p-values associated with these correlation coefficients.

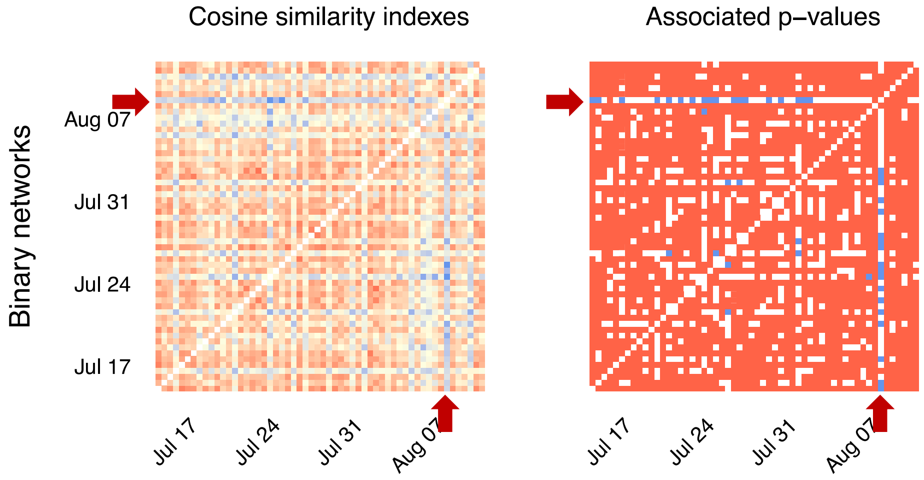

Supplementary Figure 5: Pairwise cosine similarity indexes between all possible pairs of daily binary networks (left) and associated permutation-based (right). Red arrows depict the day when raw cosine indexes were found to be significantly lower than expected by chance (August 7th, 2017, at night).

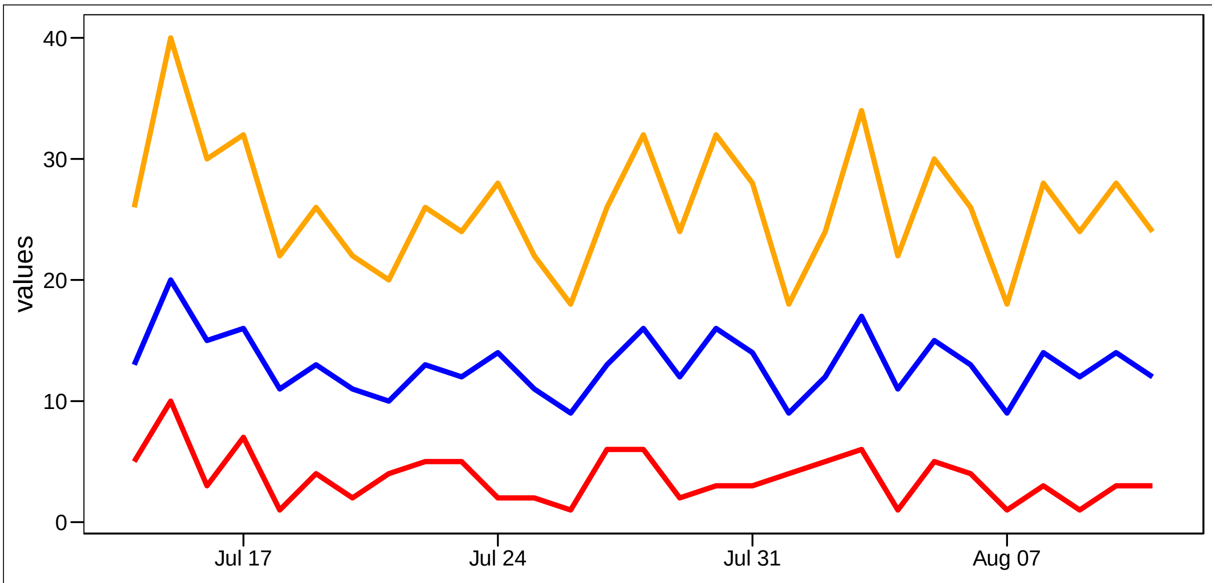

Supplementary Figure 6: Number of edges (blue), number of triads (red) and number of edges in common with the network built on the whole study period (orange).

Whether we consider the increased levels of similarity in the weighted networks or the exception of the 7th of August 2017 in the binary networks, we could not identify which ecological/behavioural factors generated such patterns. Indeed, no peak in human activity, nor predator presence, nor changes in research activities were recorded that day.

Supplementary References

1. Watson-Haigh, N. S., O'Neill, C. J. & Kadarmideen, H. N. Proximity loggers: Data handling and classification for quality control. *IEEE Sens. J.* **12**, 1611–1617 (2012).

2. Boyland, N. K., James, R., Mlynski, D. T., Madden, J. R. & Croft, D. P. Spatial proximity loggers for recording animal social networks : Consequences of inter-logger variation in performance. *Behav. Ecol. Sociobiol.* **67**, 1877–1890 (2013).

3. Drewe, J. A. *et al.* Performance of proximity loggers in recording Intra- and Inter-species interactions : A laboratory and field-based validation study. *PLoS One* **7**, (2012).

4. Crates, R. A. *et al.* Individual variation in winter supplementary food consumption and its consequences for reproduction in wild birds. *J. Avian Biol.* **47**, 678–689 (2016).

5. Prange, S., Jordan, T., Hunter, C. & Gehrt, S. D. New Radiocollars for the Detection of Proximity among Individuals. *Wildl. Soc. Bull.* **34**, 1333–1344 (2006).

6. Silk, M. J. *et al.* Seasonal variation in daily patterns of social contacts in the European badger meles meles. *Ecol. Evol.* **7**, 9006–9015 (2017).

7. Hoppitt, W. & Farine, D. Association Indices For Quantifying Social Relationships: How To Deal With Missing Observations Of Individuals Or Groups. *Anim. Behav.* **136**, 227–238 (2018).

8. Farine, D. R. asnipe: Animal Social Network Inference and Permutations for Ecologists. (2019).

9. Siegel, J. M. Do all animals sleep? *Trends Neurosci.* **31**, 208–213 (2008).

10. Wild, F. lsa: Latent Semantic Analysis. R package version 0.73.2. https://CRAN.R-project.org/package=lsa. (2020).

11. Dai, H., Leeder, J. S. & Cui, Y. A modified generalized fisher method for combining probabilities from dependent tests. *Front. Genet.* **5**, 1–10 (2014).
