# Supplementary material for: High-resolution tracking of hyrax social interactions highlights nighttime drivers of animal sociality
Source: Commun Biol. 2022 Dec 15;5:1378. doi: 10.1038/s42003-022-04317-5 (PMC9755157; doi:10.1038/s42003-022-04317-5)
Supplement: Supplementary file 3 — Reporting Summary [file 42003_2022_4317_MOESM3_ESM.pdf]

nature portfolio

Corresponding author(s): Amiyaal Ilany
COMMSBIO-22-1260A

Last updated by author(s): Nov 12, 2022

Reporting Summary

Nature Portfolio wishes to improve the reproducibility of the work that we publish. This form provides structure for consistency and transparency in reporting. For further information on Nature Portfolio policies, see our Editorial Policies and the Editorial Policy Checklist.

Statistics

For all statistical analyses, confirm that the following items are present in the figure legend, table legend, main text, or Methods section.

n/a	Confirmed	
☐	☒	The exact sample size (*n*) for each experimental group/condition, given as a discrete number and unit of measurement
☐	☒	A statement on whether measurements were taken from distinct samples or whether the same sample was measured repeatedly
☐	☒	The statistical test(s) used AND whether they are one- or two-sided<br>*Only common tests should be described solely by name; describe more complex techniques in the Methods section.*
☒	☐	A description of all covariates tested
☐	☒	A description of any assumptions or corrections, such as tests of normality and adjustment for multiple comparisons
☐	☒	A full description of the statistical parameters including central tendency (e.g. means) or other basic estimates (e.g. regression coefficient) AND variation (e.g. standard deviation) or associated estimates of uncertainty (e.g. confidence intervals)
☐	☒	For null hypothesis testing, the test statistic (e.g. *F*, *t*, *r*) with confidence intervals, effect sizes, degrees of freedom and *P* value noted<br>*Give P values as exact values whenever suitable.*
☒	☐	For Bayesian analysis, information on the choice of priors and Markov chain Monte Carlo settings
☒	☐	For hierarchical and complex designs, identification of the appropriate level for tests and full reporting of outcomes
☐	☒	Estimates of effect sizes (e.g. Cohen's *d*, Pearson's *r*), indicating how they were calculated

Our web collection on statistics for biologists contains articles on many of the points above.

Software and code

Policy information about availability of computer code

Data collection	Data were downloaded from Sirtrack E2C-171-A proximity loggers using the associated Sirtrack downloading interface.
Data analysis	Data pre-processing and analysis were performed in R v4.0.1, using original code and R packages cited in the main text. R code is available on the online Zenodo repository https://zenodo.org/badge/latestdoi/388145736.

For manuscripts utilizing custom algorithms or software that are central to the research but not yet described in published literature, software must be made available to editors and reviewers. We strongly encourage code deposition in a community repository (e.g. GitHub). See the Nature Portfolio guidelines for submitting code & software for further information.

Data

Policy information about availability of data

All manuscripts must include a data availability statement. This statement should provide the following information, where applicable:

- Accession codes, unique identifiers, or web links for publicly available datasets
- A description of any restrictions on data availability
- For clinical datasets or third party data, please ensure that the statement adheres to our policy

The datasets generated and/or analysed during the current study are available in the Zenodo repository, https://zenodo.org/badge/latestdoi/388145736.

Human research participants

Policy information about studies involving human research participants and Sex and Gender in Research.

Reporting on sex and gender	*Use the terms sex (biological attribute) and gender (shaped by social and cultural circumstances) carefully in order to avoid confusing both terms. Indicate if findings apply to only one sex or gender; describe whether sex and gender were considered in study design whether sex and/or gender was determined based on self-reporting or assigned and methods used. Provide in the source data disaggregated sex and gender data where this information has been collected, and consent has been obtained for sharing of individual-level data; provide overall numbers in this Reporting Summary. Please state if this information has not been collected. Report sex- and gender-based analyses where performed, justify reasons for lack of sex- and gender-based analysis.*
Population characteristics	*Describe the covariate-relevant population characteristics of the human research participants (e.g. age, genotypic information, past and current diagnosis and treatment categories). If you filled out the behavioural & social sciences study design questions and have nothing to add here, write "See above."*
Recruitment	*Describe how participants were recruited. Outline any potential self-selection bias or other biases that may be present and how these are likely to impact results.*
Ethics oversight	*Identify the organization(s) that approved the study protocol.*

Note that full information on the approval of the study protocol must also be provided in the manuscript.

Field-specific reporting

Please select the one below that is the best fit for your research. If you are not sure, read the appropriate sections before making your selection.

☐ Life sciences ☐ Behavioural & social sciences ☒ Ecological, evolutionary & environmental sciences

For a reference copy of the document with all sections, see nature.com/documents/nr-reporting-summary-flat.pdf

Ecological, evolutionary & environmental sciences study design

All studies must disclose on these points even when the disclosure is negative.

Study description	Longitudinal study of animal social networks. Study is permutation-based only and does not include models.
Research sample	Social data were collected using Sirtrack proximity loggers. Targeted group includes 37 adult individuals divided between two canyons. Population 1: 20 out of 21 individuals were succesfully fitted with collars. Population 2: 10 out of 16 individuals fitted with collars (3 adults disappeared, 3 could not be marked).
Sampling strategy	Adults were the only eligible individuals to receive proximity loggers due to weight requirements. The sampling strategy aimed at capturing all adults in the canyons.
Data collection	Events of co-occurrence in space (~ 60 cm) were recorded using Sirtrack proximity loggers
Timing and spatial scale	Start date: Jule 14th, 2017<br>End date: August 10th, 2017,<br>Data were collected 24/7, at the resolution of 1 second<br>Data were collected within 2 well established study sites (35,437 and 10,286 square meters, respectively)
Data exclusions	Individuals lighter than 1.8kg are usually under 2 years and still display juvenile behaviours (biased social interactions towards their mother, still determining their place in the social hierarchy). They were thus excluded from the study beforehand and collars types were selected accordingly (weight requirement of 1.8 kg). 1 social group was only partially marked and therefore excluded from the study after marking individuals. 3 additional individuals were removed from the sample before data processing as they either disappeared or died soon after the begining of the study.
Reproducibility	Data collected were events of co-occurrence in space. Social network traits were later calculated in R using well known packages for social network analysis. Details of data analysis procedure are included in the Supporting Information, and R code is available upon request for reproducibility. R code will be added to the Zenodo repository upon manuscript acceptance.
Randomization	The study population is located in the wild and does not allow for randomizations. Statistical analysis however relies on permutations designed to control for animal space use and temporality of the data.
Blinding	All individuals receive the same proximity loggers and observers had no prior knowledge on the study population. Later exclusion of individuals and other methodological choices were data-driven only.

Did the study involve field work? ☒ Yes ☐ No

Field work, collection and transport

Field conditions	Fieldwork in desertic area (middle east)<br>Rocky mountain slopes with little to no vegetation except for animal's dens and riverbed (good visibility)<br>No rainfall from May until October<br>Scarce rains and flash floods in March-April (affected the trapping period, but not the study period)<br>Temperatures between 35 and 45 degrees Celsius
Location	Ein Gedi Nature Reserve (Israel)<br>Dead Sea region<br>31.4663° N, 35.3944° E<br>altitude: -423 meters
Access & import/export	The fieldsites are located more than 1h hike from the reserve entrance. Valuable material such as fieldcope, proximity loggers and sensitive trapping equipment (e.g., needles, ketamine, RNA later etc.) were taken in and out of the canyons every day. Heavy pieces (e.g. traps, trapping bags, etc.) were concealed in the field. Biological samples and ketamine were placed inside cooling containers with ice packs at all times.
Disturbance	Animal handling was conducted to minimize handling time and stress. Handling was only performed if absolutely necessary (unmarked indivduals, adults identified to receive a collar). Traps were checked every 2 hours to free individuals which did not need handling.

Reporting for specific materials, systems and methods

We require information from authors about some types of materials, experimental systems and methods used in many studies. Here, indicate whether each material, system or method listed is relevant to your study. If you are not sure if a list item applies to your research, read the appropriate section before selecting a response.

Materials & experimental systems

n/a	Involved in the study	
☒	☐	Antibodies
☒	☐	Eukaryotic cell lines
☒	☐	Palaeontology and archaeology
☐	☒	Animals and other organisms
☒	☐	Clinical data
☒	☐	Dual use research of concern

Methods

n/a	Involved in the study	
☒	☐	ChIP-seq
☒	☐	Flow cytometry
☒	☐	MRI-based neuroimaging

Animals and other research organisms

Policy information about studies involving animals; ARRIVE guidelines recommended for reporting animal research, and Sex and Gender in Research

Laboratory animals	The study did not involve laboratory animals
Wild animals	Study species: rock hyraxes (Procavia capensis)<br>Population size: 83 individuals (including adults, juveniles and pups)<br>See detailed description of hyrax population, per study site, age group, and sex in the Supporting Information.<br>The study only retained adults hyraxes.
Reporting on sex	No sex-based analysis was performed for this study. Animals were sexed upon handling via visual inspection of genital organs.
Field-collected samples	The present study does not involve field-collected samples
Ethics oversight	Handling protocols for hyraxes in Ein Gedi Nature Reserve were approved by the Israeli Nature and Parks Authority (Permit number: 2017/41507).

Note that full information on the approval of the study protocol must also be provided in the manuscript.
